# Ceramide Imbalance and Impaired TLR4-Mediated Autophagy in BMDM of an ORMDL3-Overexpressing Mouse Model

**DOI:** 10.3390/ijms20061391

**Published:** 2019-03-20

**Authors:** Kerstin Kiefer, Josefina Casas, Roberto García-López, Rubén Vicente

**Affiliations:** 1 Laboratory of Molecular Physiology, Department of Experimental and Health Sciences, Universitat Pompeu Fabra, 08003 Barcelona, Spain; kerstin.kiefer1@gmx.de (K.K.); roberto.garcia-lopez@upf.edu (R.G.-L.); 2 Research Unit on Bioactive Molecules (RUBAM), Department of Biomedicinal Chemistry, Institute for Advanced Chemistry of Catalonia (IQAC), 08034 Barcelona, Spain; fina.casas@iqac.csic.es; 3 Center for Biomedical Research on Hepatic and Digestive Diseases (CIBEREHD), ISCIII, 28029 Madrid, Spain

**Keywords:** ORMDL3, asthma, ceramides, autophagy, macrophages

## Abstract

Increased orosomucoid-like 3 (ORMDL3) expression levels, due to single nucleotide polymorphisms (SNPs), have been associated with several inflammatory diseases, including asthma and inflammatory bowel diseases. ORMDL proteins inhibit serine palmitoyltransferase (SPT), the first rate-limiting enzyme in de novo sphingolipid synthesis and alter cellular calcium homeostasis. Both processes are essential for immune response. The present study addresses ORMDL3 protein involvement in macrophage physiology using an overexpressing knock-in mouse model. Ceramide content was notably different in the bone-marrow-derived macrophages (BMDM) from the transgenic mouse model compared with the wild type (WT) macrophages. Our data revealed an alteration of de novo production of sphinganine upon BMDM activation in the transgenic mouse. Gene-expression analysis showed that alteration in ORMDL3 expression levels did not affect activation or macrophage polarization. Nevertheless, we studied phagocytosis and autophagy—crucial processes that are dependent on lipid membrane composition. Phagocytosis in transgenic macrophages was not affected by ORMDL3 overexpression, but we did find a reduction in toll-like receptor 4 (TLR-4)-mediated autophagy. Both genetic and functional studies have pointed to autophagy as an essential pathway involved in inflammation. We believe that our work provides new insights into the functional link between ORMDL3 expression and inflammatory diseases.

## 1. Introduction

Expression of the orosomucoid-like 3 (*ORMDL3*) gene has been genetically linked to pro-inflammatory diseases, such as asthma, Crohn’s disease, ulcerative colitis, and rheumatoid arthritis [1,2,3,4], suggesting ORMDL3 involvement in immune system function. This connection has led to extensive studies seeking to identify possible underlying mechanisms. Our laboratory has previously demonstrated ORMDL3 involvement in calcium homeostasis [5], affecting store-operated calcium entry (SOCE) [6,7] and, thereby, negatively affecting T-cell activation.

ORMDL proteins have also been described as negative regulators of serine palmitoyltransferase (SPT), the rate-limiting enzyme of de novo sphingolipid synthesis [8,9], an important signaling pathway in inflammation [10]. Considering the pathophysiology previously described, the implicit impact of the disease-associated ORMDL3 in sphingolipid synthesis was initially thought to be irrelevant because neither ORMDL3 knock-down nor ORMDL3 overexpression had any effect on SPT activity in Hela and HEK293 mammalian cells lines [11,12] and transgenic mouse models [13]. However, alterations in ceramide species have been attributed to ORMDL3 expression levels in the RAW264.7 macrophage cell line [14]. Moreover, in human hepatoma HepG2 cells, downregulation of ORMDL3 expression has been shown to induce dihydroceramide production [15]. In the same model, it was shown that pro-inflammatory mediators during acute-phase response, such as IL1 and oncostatin M, modulated ORMDL expression and dihydroceramide production [15]. There is further evidence that supports the links between ORMDL3, ceramides, and inflammation. Thus, overexpression of ORMDL3, or treatments with the SPT inhibitor myriocine, promote inflammation by inducing IL6 and IL8 release in airway epithelial cells [16]. Moreover, using in vivo mouse models, it has been reported that similar approaches cause airway hyper-reactivity [17,18].

In the present study, we explore the role of ORMDL3 in innate immunity using a transgenic mouse model overexpressing the human protein; this model is able to mimic the risk allele in inflammatory diseases [4]. We focus on macrophage physiology, paying special attention to ceramide synthesis and the processes derived from it, including activation, polarization, phagocytosis, and autophagy. We have previously described how macrophage activation underlies the coordinated regulation of all three ORMDL isoforms in order to allow the induction of de novo sphingolipid synthesis [12], which is linked to important processes in macrophage physiology, such as autophagy and phagocytosis [12]. 

Altogether, our work provides new evidence of the role of ORMDL proteins in ceramide synthesis, reinforcing the idea of transcriptional regulation of *ORMDL* genes. In addition, our results demonstrate the consequences of anomalous expression of ORMDL3 on ceramide homeostasis in macrophages, which affects important processes in innate immunity, such as autophagy.

## 2. Results

### 2.1. Ceramide Composition in BMDM from hORMDL3^Rosa26^ Mice

**Figure 1:**
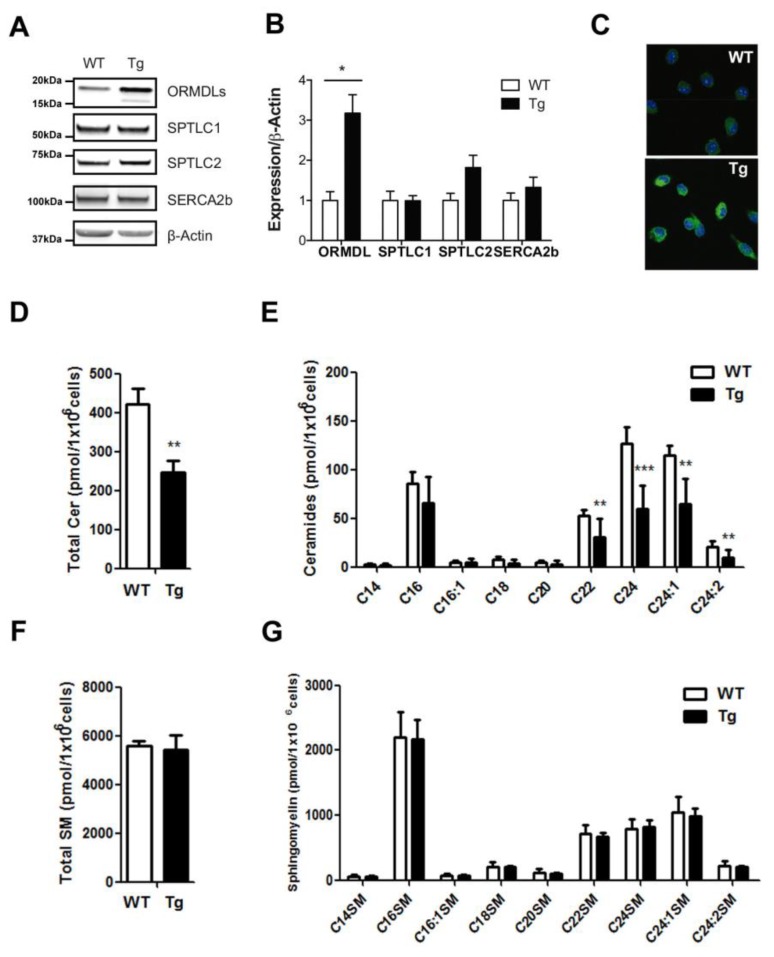
Ceramide content of macrophages in hORMDL3^Rosa26^ mice. (**A**,**B**) Western blot in wild type (WT) and transgenic (Tg) mice of SERCA2b, SPTLC1, SPTLC2, ORMDLs, and actin, using 50 µg of protein from bone-marrow-defined-macrophages (BMDM). (**A**) Representative Western blot; (**B**) graph with quantification analysis normalized to actin. Statistics: *n* = 4; Mann-Whitney test; * *p* < 0.05; (**C**) immunostaining using anti-ORMDL antibody (green) and TO-PRO-3 (blue) of BMDM from WT and Tg mice; (**D**,**E**) Ceramide content in BMDM macrophages from WT and hORMDL3^Rosa26^ (Tg) mice, quantified by mass spectrometry. (**D**) Graph with total ceramide content. (**E**) Contribution of the different ceramide species. (**F**) Graph with total sphingomyelin content. (**G**) Contribution of the different sphingomyelin species. Statistics (D–F): WT *n* = 13, Tg *n* = 11; Mann-Whitney test; * *p* < 0.05; ** *p* < 0.01; *** *p* < 0.01. Error bars represent standard error.

We isolated BMDM from our *hORMDL^Rosa26^* transgenic mice model and checked the induction of ORMDL3 protein expression. Our results, using an antibody against ORMDL proteins, showed a three-fold induction (Figure 1A,B). The double band observed for ORMDLs in transgenic animals has already been reported [9,12,13]. We also looked for the expression levels of known interactors of ORMDL3. In this context, we did not observe significant alterations in protein expression of the sarco/endoplasmic reticulum Ca^2+^-ATPase isoform 2b (SERCA2b) and of SPT components, serine palmitoyltransferase long-chain base subunit 1 and 2 (SPTLC1, SPTLC2) (Figure 1A,B). Immunostainings using anti-ORMDL antibodies confirmed the reticular expression pattern of the overexpressed protein (Figure 1C). 

We then studied the ceramide composition in BMDM from hORMDL3^Rosa26^ compared to that in WT mice (Figure 1D,E). Our results showed a significant decrease in the basal sphingolipid content (Figure 1D). Detailed analysis revealed reductions across the entire range of the ceramide species analyzed, particularly in the long-chain species C22, C24:0, C24:1 and C24:2 (Figure 1E). In the same samples, we analyzed sphingomyelin content (Figure 1F,G). We did not observe alteration in total sphingomyelin or in the contribution of the different species.

**Figure 2:**
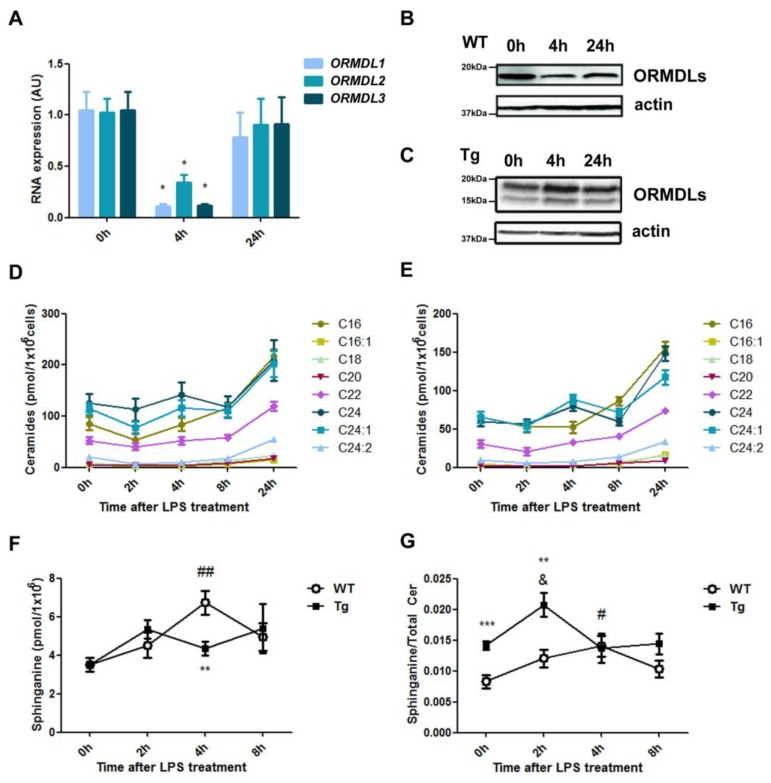
Ceramide production upon lipopolysaccharide (LPS) treatment in BMDM. **A**–**C**, Regulation of ORMDLs after activation with 100 ng/mL LPS of BMDM at indicated time points. (**A**) Gene expression analyzed by real-time PCR of ORMDL1, ORMDL2, and ORMDL3. Data are normalized to β-Actin. Statistics: *n* = 4; Kruskal-Wallis test compared to time 0; * *p* < 0.05. (**B**,**C**) Representative Western blot of ORMDL expression in WT (**B**) and Tg mice (**C**) after LPS treatment. (**D**,**E**) Content of different ceramide species in BMDM macrophages from WT (**D**) and transgenic hORMDL3^Rosa26^ (**E**) upon treatment with 100 ng/mL LPS for up to 24 h. (**F**,**G**) Sphinganine content in LPS-activated macrophages in WT and Tg BMDM. (**F**) Total sphinganine content over time. (**G**) Ratio between sphinganine content and total ceramide over time. Statistics: WT *n* = 9, Tg *n* = 7; Bonferroni ANOVA test compared to time 0 of WT BMDM (#) and Tg BMDM (&). *t*-test between WT and Tg BMDM (*) at different time points; ** *p* < 0.01; *** *p* < 0.001; # *p* < 0.05; ## *p* < 0.01; & *p* < 0.05. Error bars represent standard error.

Macrophage activation by LPS leads to an increase in intracellular ceramides [19,20,21,22], which mainly originate from the de novo pathway at early time points and from other sources later on [22,23]. We have previously demonstrated that the mechanism underlying this increase is based on a time-dependent downregulation of all three ORMDL isoforms after LPS activation in RAW264.7 macrophages [12]. Our experiments in BMDM confirmed this coordinated regulation at both the transcriptional and translational levels (Figure 2A,B). The protein expression analysis revealed a fast turnover of ORMDL proteins upon LPS activation stimulus. However, this regulation was lost in the transgenic mice in which ORMDL3 protein expression is controlled by the *Rosa26* locus (Figure 2C). In this context, we monitored, during a 24-h period, different ceramide species upon LPS stimulation in BMDM from WT (Figure 2D) and transgenic mice (Figure 2E). As expected, ceramides increased steadily over the time course of LPS activation in the WT cells, showing a marked increase at 24 h. Despite the reduced amount of ceramide content (Figure 1E), we did not observe major differences in the dynamics of ceramide induction in BMDM from hORMDL3^Rosa26^ mice (Figure 2E). We then hypothesized that stable transgenic ORMDL3 overexpression might impair the release of SPT activity and, consequently, the de novo ceramide synthesis pathway. In order to study this possibility, we monitored the amount of sphinganine over time upon LPS stimulation (Figure 2F,G). We found that sphinganine was induced at early time points in BMDM from WT animals (Figure 2F). A lesser induction was observed in BMDM from hORMDL3^Rosa26^ mice (Figure 2F). Moreover, when comparing the ratio between sphinganine and total ceramides, we were able to observe an imbalance in the membrane composition of BMDM from hORMDL3^Rosa26^ mice in basal condition and during the early stages of LPS stimulation (Figure 2G).

### 2.2. Macrophage Activation and M1/M2 Polarization in hORMDL3^Rosa26^ Mice

**Figure 3:**
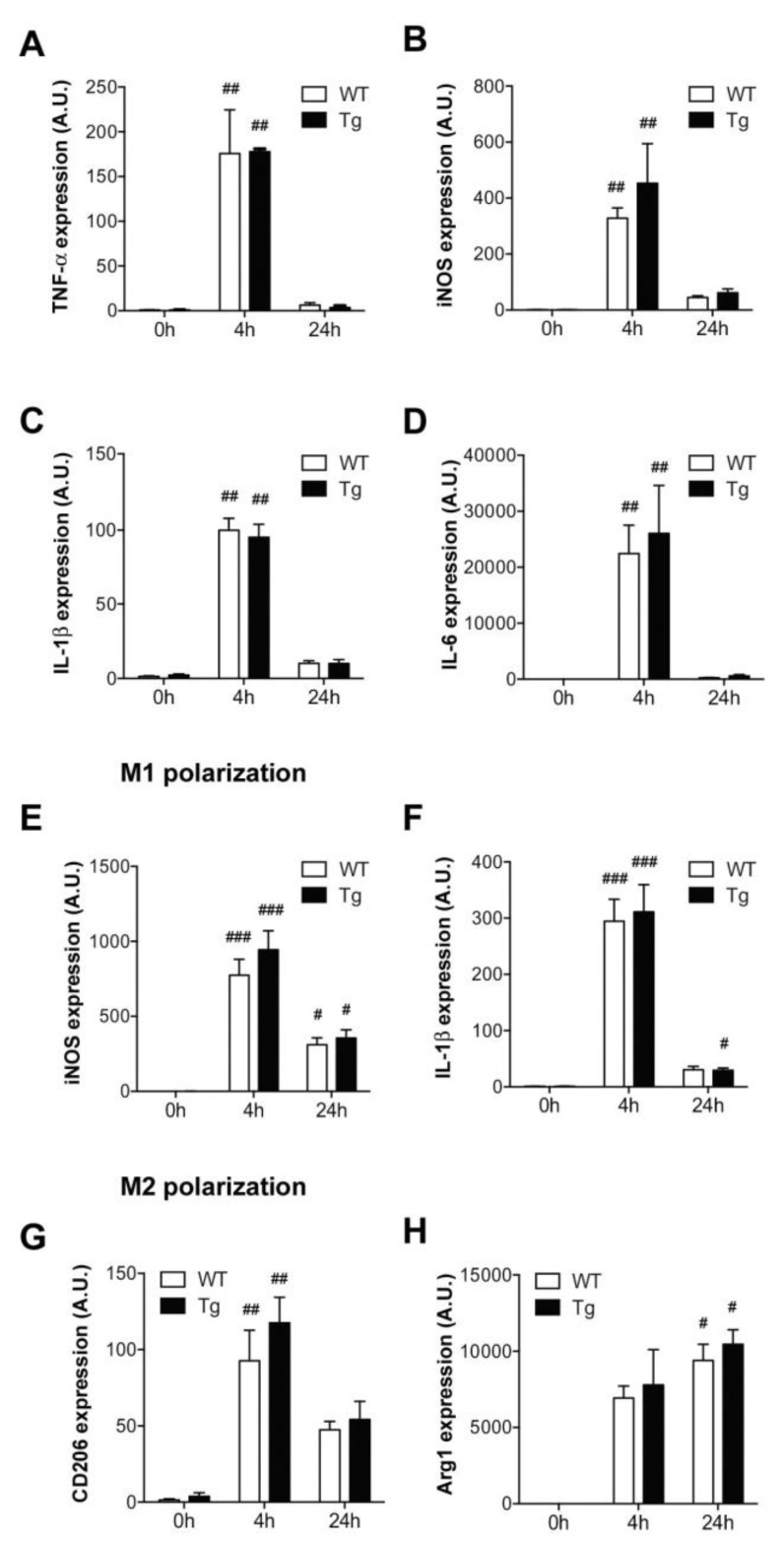
Activation profile of macrophages in hORMDL3^Rosa26^ mice. (**A**–**D**) Gene expression of activation markers analyzed by real-time PCR in BMDM macrophages from WT and Tg mice at indicated time points upon LPS (100 ng/ml) treatment. (**A**) TNF-α, (**B**) inducible nitric oxide synthase (iNOS), (**C**) IL-1β, and (**D**) IL-6. (**E**,**F**) Effect of 100 ng/mL LPS and 30 ng/mL INF-γ on M1 polarization, according to iNOS (**E**) and IL-1β (**F**) expression. (**G**,**H**) Effect of 20 ng/mL IL-4 on M2 polarization, according to CD206 (**G**) and Arg1 (**H**) expression. Data are normalized to GAPDH. Statistics: *n* = 4–8 Kruskal-Wallis test compared to time 0; # *p* < 0.05, ## *p* < 0.01; ### *p* < 0.001. Error bars represent standard error.

Macrophage activation is a crucial step in the onset of the innate immune response. In order to evaluate the impact of ORMDL3 overexpression on macrophage physiology, we studied several activation markers over time (Figure 3A–D). Our results showed that the induction and sequential regulation of TNF-α, IL1-β, IL-6 and the inducible nitric oxide synthase (iNOS) enzyme, after macrophage activation with LPS, were not altered by increased ORMDL3 expression levels.

To further explore whether ORMDL3 expression levels disturb the macrophage homeostatic function, we studied the polarization process. M1 phenotype was induced by combining LPS and INF-γ stimuli, as described in the Materials and Methods section. The expression levels of the M1 markers iNOS and IL-1β increased equally, regardless of ORMDL3 levels (Figure 3E,F). On the other hand, IL-4 and IL-13 are well-described inducers of the anti-inflammatory M2 phenotype. As expected, IL-4 alongside IL-13 treatment increased Arginase-1 (Arg1) and mannose receptor CD206 expression (Figure 3G,H). However, we observed no differences between the WT and Tg macrophages. 

### 2.3. Phagocytosis and Autophagy in hORMDL3^Rosa26^ Mice

**Figure 4:**
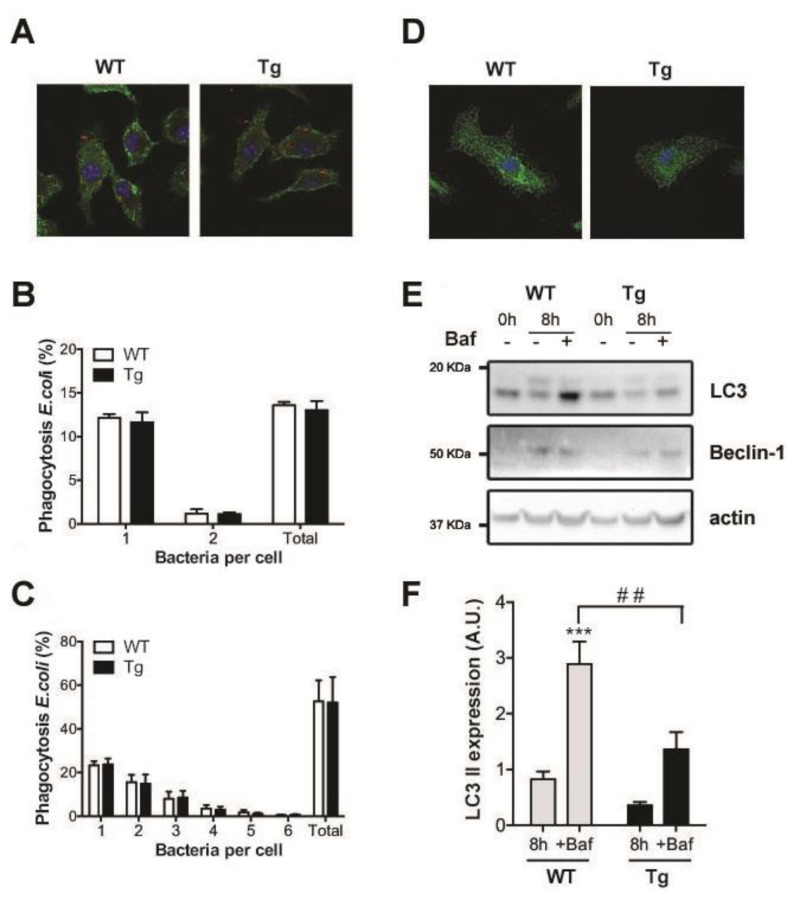
Phagocytosis and autophagy analysis in BMDM from hORMDL3^Rosa26^ mice. (**A**–**C**) Intracellular phagocytic uptake of *E. coli* after 30 min of incubation in BMDM from WT and Tg mice. (**A**) Immunofluorescence staining of fluorescently labeled *E. coli* (red) at a 5:1 ratio; Concanavalin A staining (green) marks the plasma membrane and Topro counterstain (blue) indicates the location of nuclei. Pictures were taken using a SP8 confocal microscope with a 63× objective. (**B**,**C**) Quantification of intracellular *E. coli* per cell at a ratio of (**B)** 5:1 and **C,** 20:1 displayed in % with respect to the total number of cells (*n* = 3). *(***D**–**F**) Autophagic markers after 100 ng/mL LPS treatment in BMDM from WT and hORMDL3^Rosa26^ (Tg) mice. (**D**) Immunofluorescence staining of LC3 (green) and TO-PRO-3 (*blue*) after 8 h of LPS treatment (**E**) Representative Western blot of LC3 and Beclin-1 after 8 h of LPS treatment without and with Bafilomycin A (*Baf*). (**F**) Quantification of their expression after 8 h of LPS treatment. Data have been corrected by β-Actin and normalized to values at time 0 h in each group (*n* = 6; *t*-test analysis of WT compared to untreated WT *** *p* < 0.001; *t*-test analysis of Tg compared to WT ## *p* < 0.01). Error bars represent standard error.

The alteration in membrane composition led us to explore other relevant processes in macrophage activity where cellular membranes play an important role. First, we tested possible modifications of phagocytosis in macrophages of hORMDL3^Rosa26^ mice. To monitor the phagocytic engulfment of pathogens, BMDM were treated with fluorescently tagged *E. coli* for 30 min (Figure 4A). We used different infection ratios 1:5 (Figure 4B) and 1:20 (Figure 4C). After this incubation time, we measured intracellular bacterial staining in BMDM and observed no changes in bacterial uptake between the hORMDL3^Rosa26^ and WT cells (Figure 4B,C).

Autophagy is another membrane-associated process linked to de novo sphingolipid synthesis in macrophage physiology. The activation of TLR-4 receptors in macrophages has been reported to induce autophagy [22]. We stimulated TLR-4 receptors of BMDM from hORMDL3^Rosa26^ and WT mice by incubating them with LPS for 8 h. In order to explore autophagic flux, we added Bafilomycin A (Baf). This inhibits autophagosome and lysosome fusion, and blocks LC3-II degradation and permits its accumulation. Figure 4D shows a representative image of LC3 vesicles in BMDM from both genotypes. We quantified autophagy induction by Western blot. Although Beclin-1 expression levels were similar in both genotypes, the conversion of LC3-I to LC3-II was altered (Figure 4E,F). Thus, we obtained a significant reduction of the lipidated LC3-II form in activated macrophages from Tg mice compared to those from WT mice in bafilomycin-treated samples (Figure 4F). 

## 3. Discussion

Genome-wide association studies have associated multiple single nucleotide polymorphisms (SNPs) in the *ORMDL3*-containing chromosomal region 17q21 with the risk of developing proinflammatory diseases [1,2,3]. Genetic variation at SNP rs7216389 (T-allele) increases *ORMDL3* expression, which is assumed to influence cellular physiology of the immune system, thereby contributing to associated inflammatory pathologies. Our main objective in this study was to characterize the impact of ORMDL3 on the innate immune system, focusing on macrophage physiology. This is of particular relevance because our group has previously correlated the regulation of ORMDL proteins with increased de novo ceramides during physiological macrophage activation [6]. In this work, we used the BMDM of a transgenic mouse model overexpressing hORMDL3. We decided to overexpress hORMDL3 to resemble human pathophysiology associated with the *ORMDL3* gene. Mouse and human ORMDL3 proteins share 97% sequence identity, and it has been shown that hORMDL3 function is evolutionary conserved because it is able to rescue yeast knock-out (KO) strands [9]. 

Macrophages are the first line of defense and are able to detect pathogens and cytokines expressed by other cells of the immune system. In this immune-defense scenario, two different populations of macrophages have been described, one having a proinflammatory profile (M1) and the other a homeostatic-repair function (M2). We have studied the activation profile of BMDM from transgenic mice compared with that from WT macrophages. The induction of TNFα and iNOS and the expression of proinflammatory cytokines IL-1β and IL-6 showed a similar pattern in both genotypes. This finding is in agreement with recent work performed in the RAW264.7 cell line with moderate ORMDL3 overexpression [14]. We also polarized the macrophages of the transgenic mice towards M1 and M2 profiles. The expression of M1 markers, iNOS and IL-1β, and M2 markers, CD206 and Arg1, was not different in BMDM from WT and from Tg animals. These results argue against the idea that a proinflammatory profile in macrophages is the underlying cause of the genetic association of ORMDL3 with inflammatory diseases.

ORMDL proteins have been claimed to be intracellular sphingolipid sensors and negative SPT regulators. Whether SNPs that regulate *cis*, leading to increased *ORMDL3* expression, are related to the specific function of this protein in sphingolipid synthesis remains an open question. In some studies, including our previous work [6], ORMDL3 was observed to have no impact on basal sphingolipid levels by transient transfection in different cell types [11]. By contrast, with regard to the macrophage cell line RAW264.7, it has been recently reported that higher levels of ORMDL3 have decreased sphingolipids de novo, especially C16-, C22-, and C24-backbone [14]. In addition, in an ORMDL3 KO mouse model, increased amounts of sphinganine in different tissues have been described [13]. Our results regarding BMDM from transgenic mice have demonstrated that increased ORMDL3 expression affects cellular ceramide content, confirming its importance in macrophage physiology. The reduction in rather long-chain ceramide species might be caused by a more complex scenario, in which ORMDL3 would also regulate the activity of certain ceramide synthases or ceramidases downstream of SPT, as has been previously suggested [13,14]. We did not detect alteration in the sphingomyelin content of BMDM from transgenic mice. This fact has been previously reported in other cell models in which ORMDLs expression levels have been modified [15] and would argue for an impact on specific ceramide synthesis pathways and the presence of compensatory mechanisms. The present work has explored the effect of macrophage activation on a de novo synthesis pathway in response to TLR-4 stimulation with LPS. ORMDL3 overexpression in transgenic macrophages is under the control of an exogenous promoter resisting the coordinated ORMDL family downregulation that releases the enzymatic activity (Figure 2C). This might explain the different pattern of sphinganine production upon LPS stimulation (Figure 2F). Moreover, despite the total amount of sphinganine being equivalent in BMDM from both genotypes in basal condition, the membrane composition, considering the ratio between sphinganine and ceramides, was considerably altered in macrophages from the transgenic mouse model (Figure 2G). 

We monitored two membrane-sensitive processes: phagocytosis and autophagy. Myriocin treatment is known to inhibit phagocytosis of fungal *Candida albicans* [24]. In our model, however, phagocytosis was not impaired by ORMDL3 expression after a pathogenic *E. coli* incubation. In a similar scenario, autophagy induction after LPS exposure was seen to be highly dependent on de novo ceramide synthesis [22]. In our case, increased ORMDL3 expression impaired autophagy induction, particularly at the level of autophagosome elongation, as LC3-II was reduced in the transgenic mouse. It has been previously reported that an up-regulation of ORMDL3 is required to promote autophagy in endothelial cells upon ox-LDL treatment [25]. Moreover, in heterologous expression systems, it has been shown that ORMDL3 expression modulates autophagic flux induced by mTOR1 inhibition [26]. Autophagy is a process induced by different pathways that determine cell fates in different ways. The blockage of autophagic flux, observed in our work, was likely caused by the impact that ORMDL3 had on the de novo synthesis pathway, an essential step in the autophagy induced by LPS. Our data also suggest that the alteration observed in the Tg mice would be independent of Beclin-1. Dysregulation of autophagy after bacterial infection might lead to a negative outcome of bacterial clearance, leading to sustained inflammation [27]. Indeed, anomalous autophagy has been linked to inflammatory bowel diseases [28,29], a spectrum of inflammatory disorders to which *ORMDL3* has been genetically associated [1,2,30].

The present study has addressed the role of ORMDL3 in innate immunity in an effort to better understand the link between a propensity to develop inflammatory diseases and *cis* regulatory elements around *ORMDL3*. Our work reinforces the current view of ORMDLs as being negative modulators of SPT activity and having special importance to macrophage physiology. Following macrophage activation, our transgenic mouse model showed reduced ceramide content and modifications in de novo synthesis, together with impaired autophagy. An alteration in autophagy could contribute to the risk of inflammatory diseases, suggesting an attractive area for further research.

## 4. Materials and Methods

### 4.1. hORMDL3^Rosa26^ Mouse Generation

A transgenic (Tg) mouse model that ubiquitously overexpressed hORMDL3 in a C57BL/6 background mouse line was generated. The gene was inserted within the endogenous *Rosa26* locus. Animal procedures (JMC-07-1001P3) were approved (02/06/14) by the ethics committee of the Barcelona Biomedical Research Park (CEEA PRBB) and by the Generalitat de Catalunya. In this work, we compared bone-marrow-derived macrophages (BMDM) from transgenic animals (Tg) to that from littermates that did not have the transgene (WT).

### 4.2. Murine Bone-Marrow-Derived Macrophages Isolation and Differentiation

Bone-marrow cells were obtained by flushing the femurs from six- to eight-week-old mice and differentiated (7 days) in Dulbecco modified Eagle medium (DMEM, Sigma-Aldrich, St. Louis, MO, USA) supplemented with 30% L929 supernatant containing macrophage-colony stimulating factor (M-CSF), 20% heat-inactivated fetal calf serum, and antibiotics [31,32].

### 4.3. In-Vitro Activation and Polarization

Adherent BMDM were activated through the TLR-4 receptors by incubation of 100 ng/mL lipopolysaccharide (LPS) for the indicated time points. For autophagy experiments, BMDM were additionally incubated with 0.1 μg/mL Bafilomycin A (Sigma-Aldrich) during the last two hours. Protein lysates were analyzed by Western blot. For M1 macrophage induction, cells were exposed to 100 ng/mL LPS and 30 ng/mL INFγ for 24 h. To obtain M2 macrophages, BMDM were treated with 10 ng/mL IL-4 and 10 ng/mL IL-13 for 24 h.

### 4.4. Quantitative Real-Time (RT) Polymerase Chain Reaction (PCR) Analysis

Total RNA of BMDM was extracted using the Nucleospin RNA II kit (Macherey-Nagel, Bethlehem, PA, USA). Quantitative RT-PCR was performed with SYBR Green (Applied Biosystems, Foster City, CA, USA). The primers used were: 5’-CTGCTGAGCATTCCCTTTGT-3’ 5’-CACGGTGTGCAGAAAGATGT-3’ ORMDL3, 5’-GACCCTCACACTCAGATCATCTTC-3’ 5’-CGCTGGCTCAGCCACTCC-3’ TNF-α, 5’-GCCTTCTTGGGACTGATGCT-3’ 5’-TGCCATTGCACAACTCTTTTC-3’ IL-6, 5’-TGCCACCTTTTGACAGTGATG-3’ 5’-AAGGTCCACGGGAAAGACAC-3’ IL-1β, 5’-TCACCTTCGAGGGCAGCCGA-3’ 5’-TCCGTGGCAAAGCGAGCCAG-3’ iNOS, 5’-GATTATCGGAGCGCCTTTCT-3’ 5’-CCACACTGACTCTTCCATTCTT-3’ Arg1, 5’-CTGCAGATGGGTGGGTTATT-3’ 5’-GGCATTGATGCTGCTGTTATG-3’ CD206, 5’-TGGAATCCTGTGGCATCCATGAAAC-3’5’-TAAAACGCAGCTCAGTAACAGTCCG-3’ β-Actin,5’-TGTCGTGGAGTCTACTGGTGTCTT-3’ 5’-TGGCTCCACCCTTCAAGTG-3’ GAPDH. In all cases, PCR conditions were 95 °C for 5 min, 95 °C for 30 s, 60 °C for 30 s, 72 °C for 30 s, and 72 °C for 5 min with 40 cycles of amplification.

### 4.5. Ceramide Quantification

A previously described cell preparation for basal lipid quantification [12] and lipid extraction and processing techniques [33] was used. Lipid analysis was done by ultraperformance liquid chromatography coupled with time-of-flight mass spectrometry in positive electrospray ionization mode, with instrument settings as described in previous studies [33] used. In the case of sphinganine, a different approach was followed. The liquid chromatography-mass spectrometer included a Waters Aquity UPLC system and Xevo TQ-S mass spectrometer (Waters, Millford, MA, USA), and operated in positive electrospray ionization (ESI) mode. The analytical column was a C8 Acquity UPLC BEH (100 mm × 2.1 mm i.d., 1.7 µm particle size, Waters). The two mobile phases were A, methanol, and B, water; both contained 0.2% (*v*/*v*) formic acid and 2 mM ammonium formate. A linear gradient was programmed—0.0 min: 20% B; 3 min: 10% B; 6 min: 10% B; 8 min: 1% B; 12 min: 1% B; 13 min: 20% B; 15 min: 20% B. The flow rate was 0.3 mL/min. The column was held at 30 °C. ESI conditions were optimized by using the following: positive ion mode, capillary voltage 3.0 kV, source temperature 150 °C, desolvation gas 1000 L/h, and desolvation temperature 450 °C. To ensure maximal sensitivity, a two-segment scan individually monitored compound transitions.

### 4.6. Western Blot

Total protein was immunodetected using rabbit antibodies against ORMDLs (1:1000), SPTLC1 (1:1000), SPTLC2 (1:1000), SERCA2b (1:1000), microtubule associated protein 1 light chain 3 beta, LC3 (1:500), Beclin-1 (1:500), and mouse anti-β-Actin (1:3000), all from Abcam, (Cambridge, UK). The antibody anti-ORMDLs (ab107639) recognizes human and mouse proteins. Secondary antibodies that were used were horseradish peroxidase-conjugated anti-rabbit and anti-mouse IgG (1:3000; GE Healthcare, Chicago, IL, USA). 

### 4.7. Phagocytosis and Fluorescence Microscopy

Macrophage infection was performed using a red fluorescent-tagged *E. coli* strain and phagocytosis assays were carried out for 30 min at 37 °C and 5% CO_2_ at a multiplicity of infection (MOI) of 1:5 and 1:20. Cells were then rinsed with ice-cold phosphate-buffered saline (PBS) and incubated with Concanavalin (Molecular Probes, Eugene, OR, USA) for 20 min on ice. After treatment, cells were fixed with 4% paraformaldehyde (PFA) for 15 min, permeabilized with 0.1% Triton-X100 and immunostained. Nuclear staining was obtained incubating permeabilized cells with TO-PRO-3 (Invitrogen, Carlsbad, CA, USA) for 10 min. Images were taken on a Leica TSC SP confocal microscope. Phagocytosed bacteria per cell were counted and compared to non-infected cells.
